# A novel nomogram and risk stratification system predicting the cancer-specific survival of patients with gastric neuroendocrine carcinoma: a study based on SEER database and external validation

**DOI:** 10.1186/s12876-023-02875-4

**Published:** 2023-07-14

**Authors:** Xue Song, Yangyang Xie, Yafang Lou

**Affiliations:** 1 Department of Respiratory and Critical Care Medicine, Hangzhou TCM Hospital, Zhejiang Chinese Medical University, #453, Tiyuchang Road, Xihu District, Hangzhou, 310000 Zhejiang province China; 2 Department of General Surgery, Hangzhou TCM Hospital Affiliated to Zhejiang Chinese Medical University, Hangzhou, 310000 Zhejiang province China

**Keywords:** Gastric cancer, SEER program, Nomogram, Risk stratification, AJCC (TNM) staging system

## Abstract

**Background:**

Gastric neuroendocrine carcinoma (GNEC) is a rare histology of gastric cancer. The retrospective study was designed to construct and validate a nomogram for predicting the cancer-specific survival (CSS) of postoperative GNEC patients.

**Methods:**

Data for 28 patients from the Hangzhou TCM Hospital were identified as the external validation cohort. A total of 1493 patients were included in the SEER database and randomly assigned to the training group (1045 patients) and internal validation group (448 patients). The nomogram was constructed using the findings of univariate and multivariate Cox regression studies. The model was evaluated by consistency index (C-index), calibration plots, and clinical net benefit. Finally, the effect between the nomogram and AJCC staging system was compared by net reclassification index (NRI) and integrated discrimination improvement (IDI).

**Results:**

Age, gender, grade, T stage, N stage, metastasis, primary site, tumor size, RNE, and chemotherapy were incorporated in the nomogram. The C-indexes were 0.792 and 0.782 in the training and internal verification sets. The 1-, 3-, and 5-year CSS predicted by the nomogram and actual measurements had good agreement in calibration plots. The 1-, 3-, and 5-year NRI were 0.21, 0.29, and 0.37, respectively. The 1-, 3-, and 5-year IDI values were 0.10, 0.12, and 0.13 (P < 0.001), respectively. In 1-, 3-, and 5-year CSS prediction using DCA curves, the nomogram outperformed the AJCC staging system. The nomogram performed well in both the internal and external validation cohorts.

**Conclusion:**

We developed and validated a nomogram to predict 1-, 3-, and 5-year CSS for GNEC patients after surgical resection. This well-performing model could help doctors enhance the treatment plan.

## Introduction

Gastric cancer (GC) is a highly heterogeneous malignancy with several pathologies exhibiting markedly varied molecular patterns, tumor behavior, and prognoses [1]. Gastric neuroendocrine carcinoma (GNEC) is a rare histological type of GC that affects 0.1–0.6% of all patients and has been on the rise in recent decades [2]. GNEC is made up of poorly differentiated endocrine cells, which present poor prognoses and tendencies to spread, and bleeding is an important cause of patient mortality [3, 4]. Lin et al. indicated that the survival of GNEC patients was much shorter than gastric adenocarcinoma [5]. Radical surgery, as the primary treatment for GNEC, is the only possible cure method. However, compared with GC patients, the prognoses of postoperative GNEC patients remained unsatisfying [6]. Therefore, determining postoperative prognostic variables is crucial for selecting treatment modalities and surveillance plans.

American Joint Committee on Cancer (AJCC) TNM staging system has played an essential role in predicting the prognosis of GNEC [7, 8]. However, other variables that are not considered by the AJCC staging system, such as age, gender, tumor location, size, grade, and treatment, may also have an influence on the prognosis in GNEC patients [9–11]. For this reason, developing individualized treatment plans and predicting the prognosis of high-risk patients require a more trustworthy model in conjunction with effective clinicopathological aspects. Due to its simplicity and intuitiveness, nomogram, a graphical calculation model with continuous scales to assess the likelihood of a specific result, has demonstrated a more accurate predictive ability than conventional staging systems [12–14]. However, no similar research has been proposed to predict postoperative survival in GNEC patients.

Based on the Surveillance, Epidemiology, and End Results (SEER) database, the study was directed to establish and validate a nomogram with a new risk stratification system for predicting the cancer-specific survival (CSS) of postoperative GNEC patients. The model’s performance was also compared with the AJCC 8th staging system.

## Materials and methods

### Data source and patient selection

The data was retrieved from the SEER database during 2010–2015 and analyzed retrospectively. External validation data was obtained from the Hangzhou TCM Hospital between January 2012 and December 2016. This research was carried out in line with the Helsinki Declaration. Participation in this study did not need written informed consent, as required by national law and institutional norms. The Ethics Committee of the Hangzhou TCM Hospital examined and approved the experiment involving human participants.

**Fig. 1:**
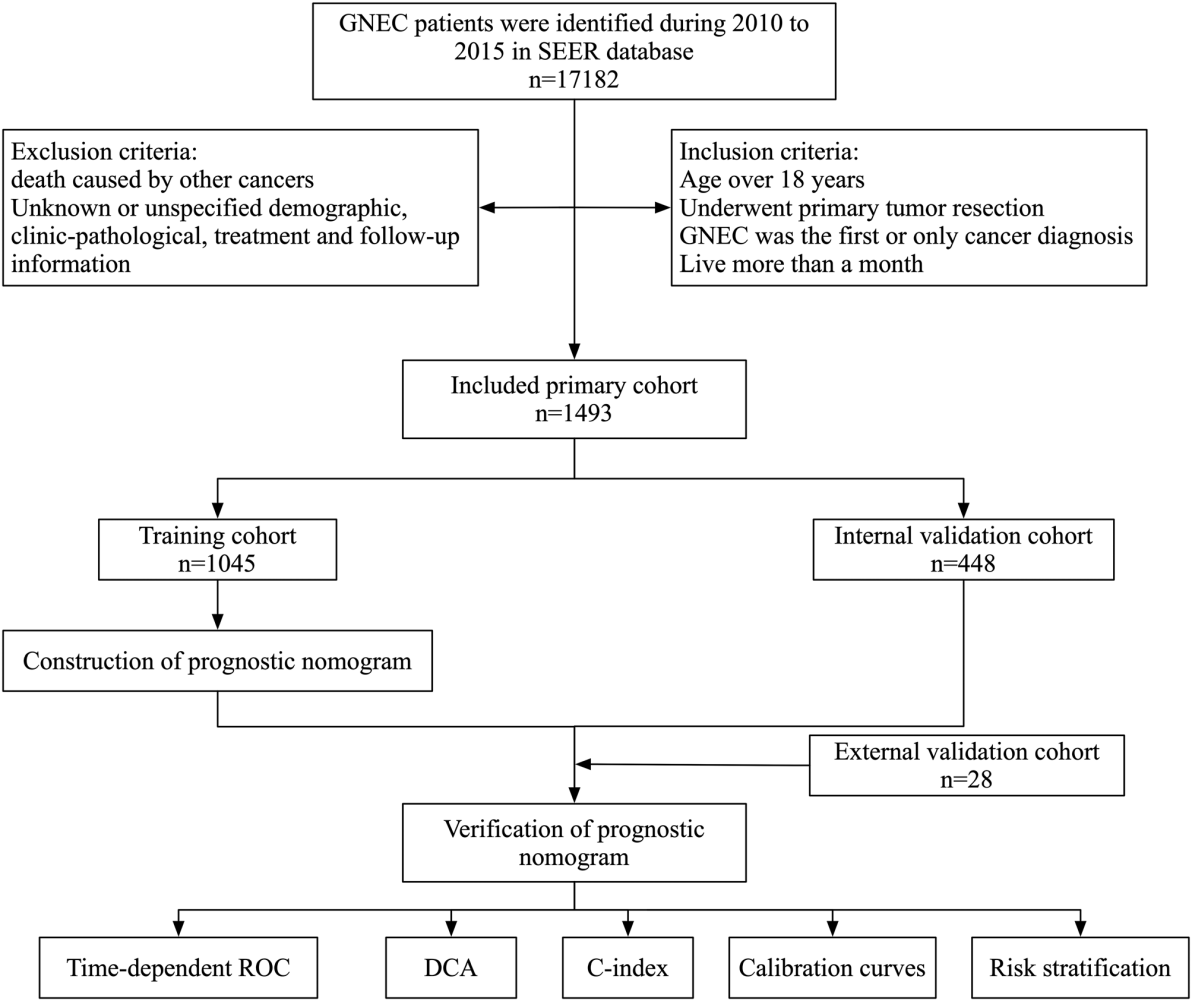
Flow diagram of the selection of eligible GNEC patients

The third edition of the International Classification of Diseases for Oncology (ICD-O-3) was used to identify cases of GNEC. 8012/3, 8013/3, 8041/3, 8042/3, 8043/3, 8044/3, 8244/3, and 8246/3 were the histological codes. The inclusion criteria were as follows: [1] older than 18 years old; [2] GNEC was the only cancer diagnosis; [3] a history of primary tumor resection; [4] the survival time was more than one month. Patients with incomplete clinicopathological information, treatment, and unknown duration of survival were excluded from the research. The detailed selection process was shown in Fig. 1.

### Clinicopathological variables

The following characteristics were extracted from the SEER database: year of diagnosis, gender, age, race, marital status, grade, AJCC stage, T stage, N stage, M stage, primary site, tumor size, regional nodes examined (RNE), surgery, radiation therapy, chemotherapy, survival time and vital status. Patients were categorized according to the primary site (cardia, distal site, middle site, and overlapping/NOS), tumor size (≤ 2 cm, ≤5 cm, and >5 cm), and RNE (0, 1–15, and ≥ 16). The GNEC patients were converted to the 8th edition of the AJCC TNM staging system based on the 7th edition in the SEER database. In the research, CSS was regarded as the endpoint. It was defined as the period from diagnosis to cancer-related mortality.

### Statistical analysis

All patients were randomized into two groups with a 7:3 ratio. The training group was employed to construct the nomogram, while the internal and external validation groups were applied to validate it. Both univariate and multivariate Cox regressions were performed to calculate the significant parameters (P < 0.05). A predictive nomogram was then established to calculate the 1-, 3-, and 5-year CSS for postoperative GNEC cases.

To assess discriminative capabilities, the consistency index (C-index) and the time-dependent area under the curve (AUC) were computed using bootstrapping. The higher value of the C-index and AUC denoted greater predictive ability. The 1-, 3-, and 5-year calibration plots (1,000 bootstrap resamples) were used to compare the predicted CSS with what was actually observed. The ideal prediction was shown to be the 45-degree line.

The net benefit for a set of threshold probabilities was calculated using decision curve analysis (DCA), which allowed researchers to test how well the model would function as a clinical decision-making tool. The optimum risk score cutoff value was utilized to create a novel risk stratification approach that divides patients into low-, middle-, and high-risk groups using the X-tile software. To compare the variations in CSS among patients in various risk stratification groups, Kaplan-Meier (KM) curves and log-rank tests were used. The C-index, Net Reclassification Index (NRI), Integrated Discrimination Improvement (IDI), and DCA were used to evaluate the new model’s enhanced predictive potential and effectiveness.

All statistical computations and visualizations were done using R software version 4.1.2 (http://www.r-project.org). A two-tailed P < 0.05 was statistically significant.

## Results

### Clinicopathological characteristics

1493 GNEC patients were enrolled and divided into the training group (1045 patients) and the internal validation group (448 patients). The 28 patients from the Hangzhou TCM Hospital who made up the external validation cohort were identified. The demographic and clinical characteristics of the patients from the SEER database were shown in Table 1. The two groups did not substantially vary in demographic or clinical factors (all P > 0.05).

**Table 1:** The basic characteristics of GNEC patients in the training and validation group

Characteristics	All	Training	Validation	P value
	N = 1493	N = 1045	N = 448	
Year at diagnosis				0.242
2010 − 1012	747 (50.0%)	512 (49.0%)	235 (52.5%)	
2013–2015	746 (50.0%)	533 (51.0%)	213 (47.5%)	
Age				0.223
≤60	424 (28.4%)	307 (29.4%)	117 (26.1%)	
>60	1069 (71.6%)	738 (70.6%)	331 (73.9%)	
Gender				0.315
Female	576 (38.6%)	394 (37.7%)	182 (40.6%)	
Male	917 (61.4%)	651 (62.3%)	266 (59.4%)	
Race				0.059
White	863 (57.8%)	587 (56.2%)	276 (61.6%)	
Non-White	630 (42.2%)	458 (43.8%)	172 (38.4%)	
Marital status				0.066
Married	916 (61.4%)	659 (63.1%)	257 (57.4%)	
Unmarried	577 (38.6%)	386 (36.9%)	191 (42.6%)	
Grade				0.626
I	258 (17.3%)	183 (17.5%)	75 (16.7%)	
II	645 (43.2%)	451 (43.2%)	194 (43.3%)	
III	560 (37.5%)	387 (37.0%)	173 (38.6%)	
IV	30 (2.0%)	24 (2.3%)	6 (1.3%)	
AJCC stage				0.854
I	486 (32.6%)	336 (32.2%)	150 (33.5%)	
II	454 (30.4%)	321 (30.7%)	133 (29.7%)	
III	436 (29.2%)	309 (29.6%)	127 (28.3%)	
IV	117 (7.8%)	79 (7.6%)	38 (8.5%)	
T stage				0.833
T1	437 (29.3%)	304 (29.1%)	133 (29.7%)	
T2	264 (17.7%)	191 (18.3%)	73 (16.3%)	
T3	488 (32.7%)	338 (32.3%)	150 (33.5%)	
T4	304 (20.4%)	212 (20.3%)	92 (20.5%)	
N stage				0.509
N0	767 (51.4%)	537 (51.4%)	230 (51.3%)	
N1	317 (21.2%)	213 (20.4%)	104 (23.2%)	
N2	202 (13.5%)	148 (14.2%)	54 (12.1%)	
N3	207 (13.9%)	147 (14.1%)	60 (13.4%)	
Metastasis				0.615
M0	1376 (92.2%)	966 (92.4%)	410 (91.5%)	
M1	117 (7.8%)	79 (7.6%)	38 (8.5%)	
Primary site				0.619
Cardia	140 (9.4%)	98 (9.4%)	42 (9.4%)	
Distal site	574 (38.4%)	407 (38.9%)	167 (37.3%)	
Middle site	548 (36.7%)	373 (35.7%)	175 (39.1%)	
Overlapping/NOS	231 (15.5%)	167 (16.0%)	64 (14.3%)	
Tumor size				0.279
≤2 cm	427 (28.6%)	304 (29.1%)	123 (27.5%)	
≤5 cm	604 (40.5%)	409 (39.1%)	195 (43.5%)	
>5 cm	462 (30.9%)	332 (31.8%)	130 (29.0%)	
RNE				0.765
>16	701 (47.0%)	485 (46.4%)	216 (48.2%)	
0	153 (10.2%)	110 (10.5%)	43 (9.6%)	
1–15	639 (42.8%)	450 (43.1%)	189 (42.2%)	
Radiation				0.367
None	1170 (78.4%)	826 (79.0%)	344 (76.8%)	
Yes	323 (21.6%)	219 (21.0%)	104 (23.2%)	
Chemotherapy				0.967
None	906 (60.7%)	635 (60.8%)	271 (60.5%)	
Yes	587 (39.3%)	410 (39.2%)	177 (39.5%)	

### Univariate and multivariate cox regression analysis

The univariate Cox regression performed in the training group indicated that age, gender, grade, T stage, N stage, metastasis, primary site, tumor size, RNE, and chemotherapy were significant prognostic parameters for GNEC patients (P < 0.05). The important variables identified by univariate Cox regression were then incorporated in multivariate Cox regression analysis, demonstrating that each of these components was an independent variable (Table 2).

**Table 2:** Univariate and multivariate Cox regression analysis of CSS for variable in GNEC patients after surgical resection

Characteristics	Univariate analysis			Multivariate analysis		
	HR	95%CI	P value	HR	95%CI	P value
Age						
≤60	Reference			Reference		
>60	1.34	1.06–1.68	0.014	1.53	1.19–1.96	0.001
Gender						
Female	Reference			Reference		
Male	1.48	1.19–1.84	< 0.001	1.32	1.06–1.65	0.014
Race						
White	Reference					
Non-White	0.95	0.78–1.17	0.65			
Marital status						
Married	Reference					
Unmarried	1.11	0.9–1.36	0.336			
Grade						
I	Reference			Reference		
II	4.07	2.5–6.63	< 0.001	1.96	1.17–3.28	0.011
III	6.94	4.28–11.26	< 0.001	2.27	1.34–3.84	0.002
IV	12.02	6.12–23.6	< 0.001	3.8	1.86–7.74	< 0.001
T stage						
T1	Reference			Reference		
T2	1.46	0.93–2.29	0.099	1.05	0.66–1.68	0.839
T3	4.82	3.41–6.81	< 0.001	2.12	1.4–3.2	< 0.001
T4	8.91	6.27–12.67	< 0.001	3.32	2.12–5.19	< 0.001
N stage						
N0	Reference			Reference		
N1	2.77	2.07–3.71	< 0.001	1.89	1.37–2.6	< 0.001
N2	4.82	3.61–6.44	< 0.001	2.59	1.85–3.65	< 0.001
N3	7.98	6.04–10.54	< 0.001	4.41	3.13–6.21	< 0.001
Metastasis						
M0	Reference			Reference		
M1	3.57	2.71–4.69	< 0.001	2.55	1.87–3.47	< 0.001
Primary site						
Cardia	Reference			Reference		
Distal site	0.62	0.45–0.84	0.002	0.52	0.37–0.73	< 0.001
Middle site	0.52	0.38–0.72	< 0.001	0.56	0.39–0.79	0.001
Overlapping/NOS	0.45	0.31–0.67	< 0.001	0.38	0.25–0.59	< 0.001
Tumor size						
≤2 cm	Reference			Reference		
≤5 cm	3.99	2.78–5.73	< 0.001	1.63	1.08–2.46	0.019
>5 cm	7.1	4.97–10.15	< 0.001	1.99	1.29–3.08	0.002
RNE						
>16	Reference			Reference		
0	0.31	0.19–0.52	< 0.001	1.77	1.01–3.09	0.046
1–15	1.05	0.85–1.28	0.672	1.63	1.31–2.03	< 0.001
Radiation						
No	Reference			Reference		
Yes	1.4	1.12–1.75	0.003	0.93	0.71–1.22	0.594
Chemotherapy						
No	Reference			Reference		
Yes	2.05	1.68–2.51	< 0.001	0.67	0.52–0.87	0.002

### Development and internal validation of the nomogram

**Fig. 2:**
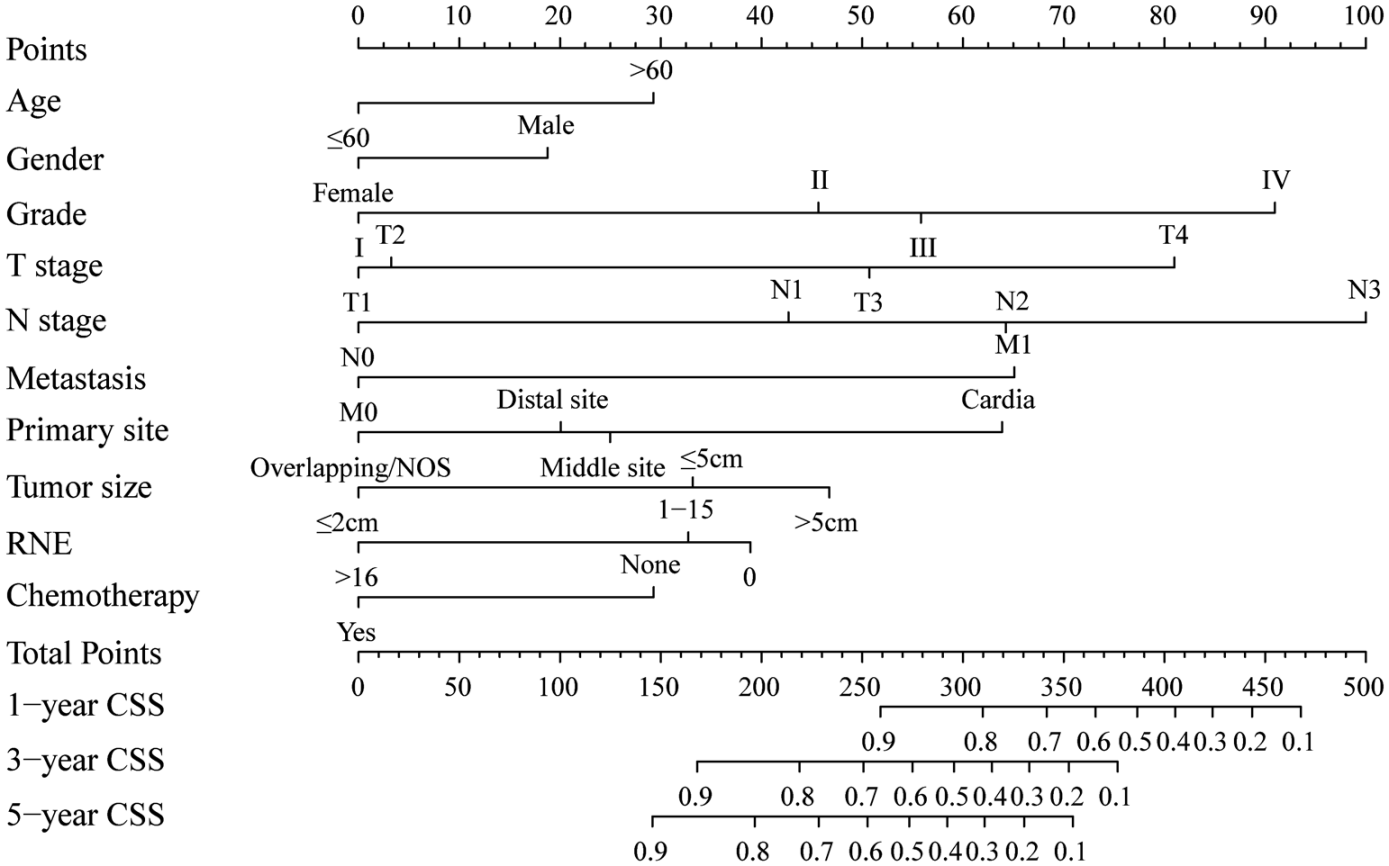
A predictive nomogram for postoperative GNEC patients

Finally, ten variables screened by multivariate Cox regression were applied to develop the nomogram for predicting the 1-, 3-, and 5-year CSS in GNEC patients (Fig. 2). The patient’s overall risk score was calculated by summing the relevant scores of each risk factor at various levels, representing the patient’s likelihood of CSS.

**Fig. 3:**
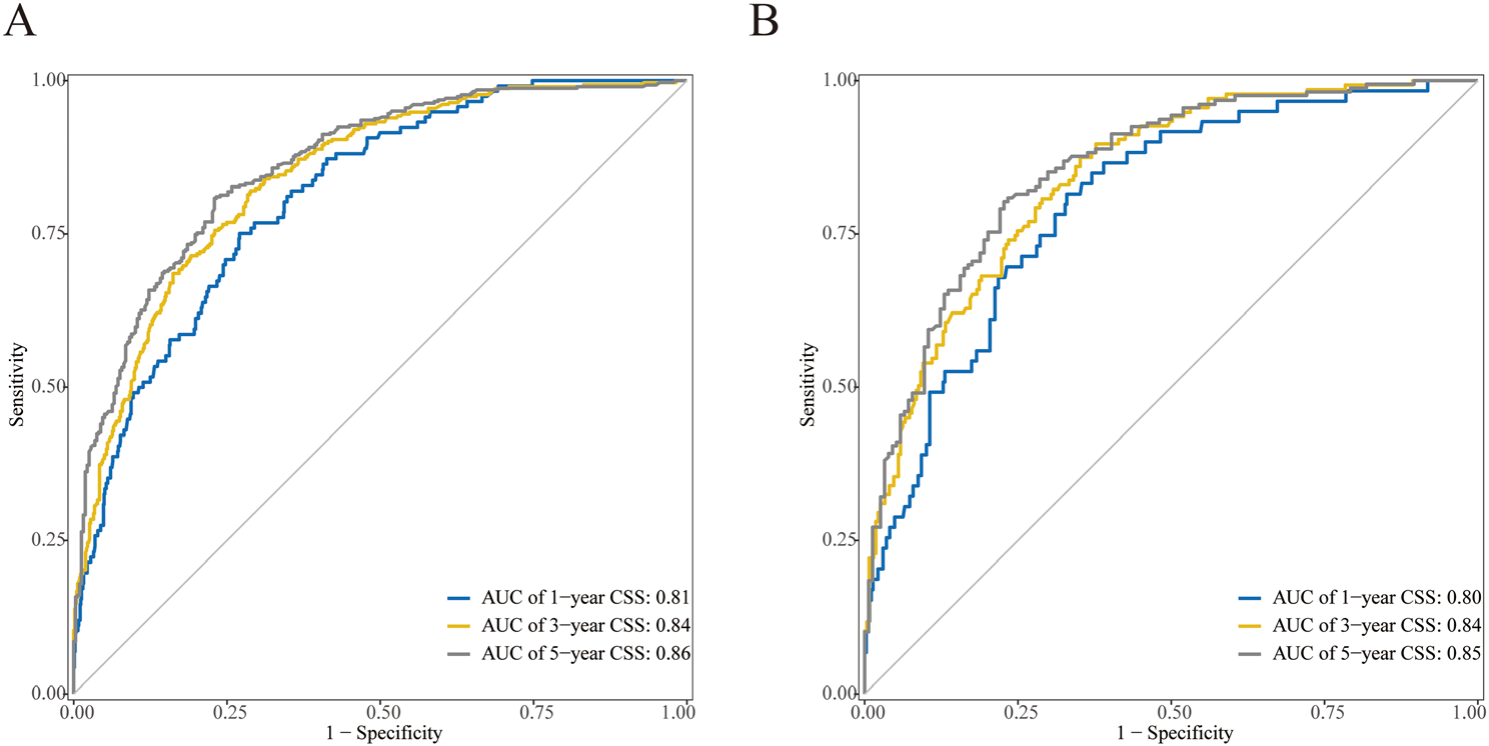
ROC of the nomogram for 1-, 3-, and 5-year CSS prediction. **(A)** Training cohorts; **(B)** Internal validation cohorts

**Fig. 4:**
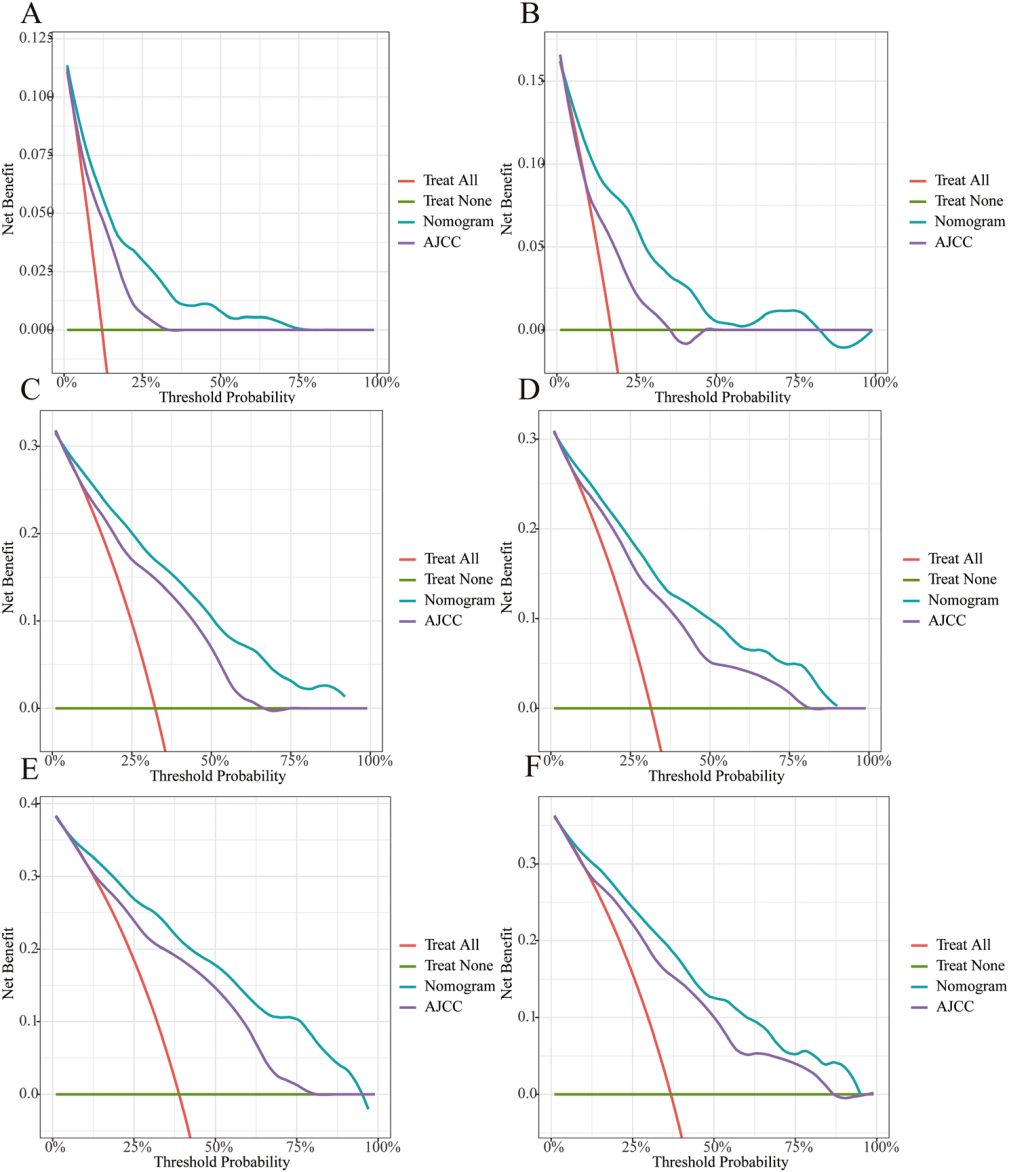
Calibration curves of 1-, 3- and 5-year CSS. **(A)** Training cohort; **(B)** Internal validation cohort

**Fig. 5:**
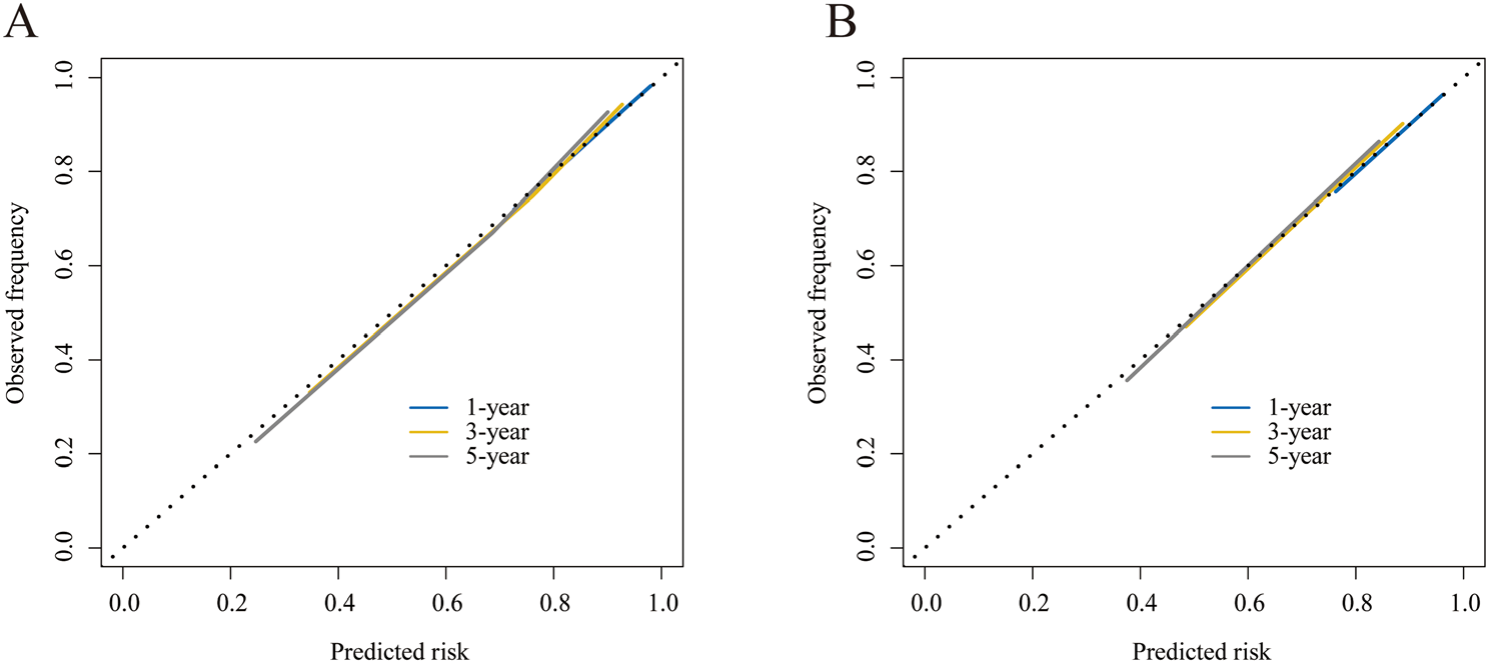
Decision curve analysis of 1-, 3- and 5-year CSS. **(A, C, E)** Training cohort; **(B, D, F)** Internal validation cohort

The C-indexes were 0.792 (95% CI: 0.770–0.813) and 0.782 (95% CI: 0.749–0.815), respectively, for the training and internal validation groups. The DCA, calibration, and receiver operating characteristic (ROC) curves were displayed in Figs. 3, 4 and 5. The ROC curves demonstrated that the nomogram exhibited excellent prediction performance (1-, 3-, and 5-year AUC for the training group were 0.81, 0.84, and 0.86; and 1-, 3-, and 5-year AUC for the internal validation cohort were 0.80, 0.84, and 0.85; Fig. 3). Additionally, outstanding therapeutic applications and good positive net benefits were demonstrated by the DCA plots at 1-, 3-, and 5-year in both groups (Fig. 4).

**Fig. 6:**
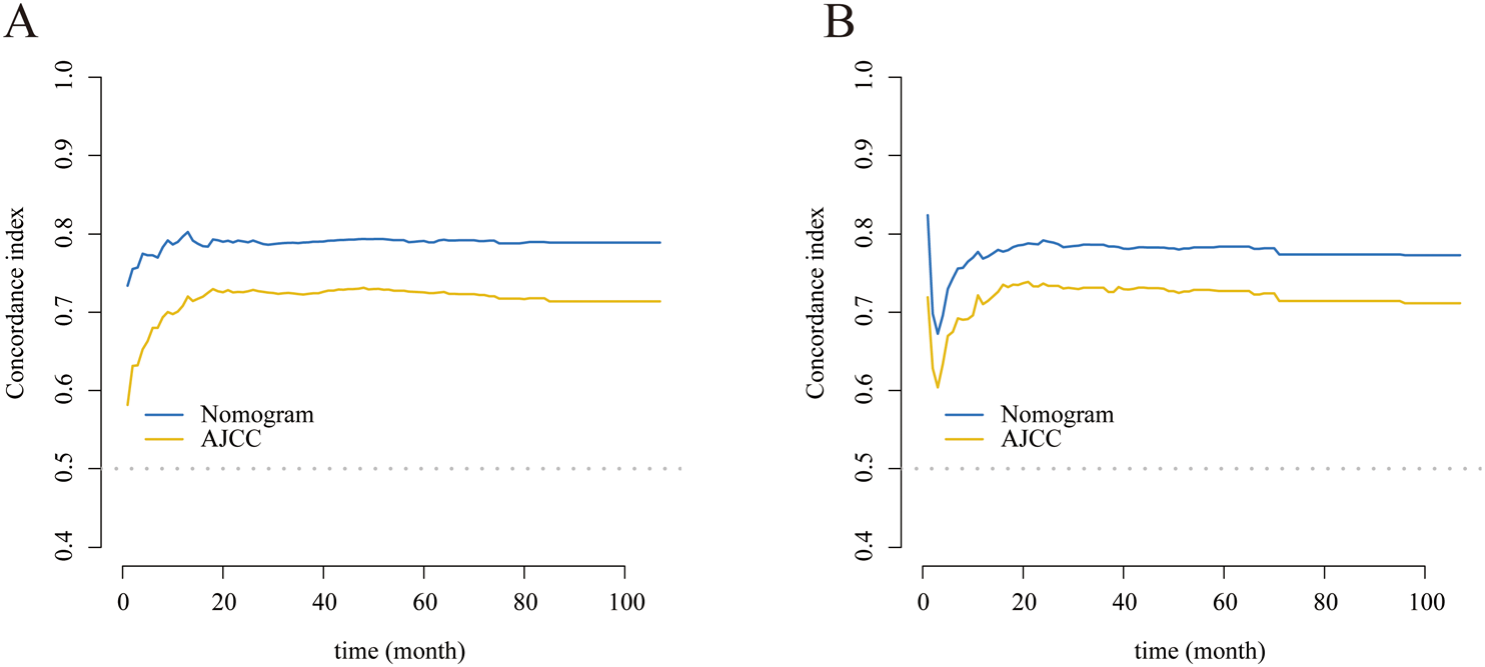
C-index analysis. **(A)** The nomogram related C-index; **(B)** AJCC staging system related C-index

The projected CSS rates at 1-, 3-, and 5-year were highly consistent with the results as shown by the calibration curves (Fig. 5). With the use of the C-index, NRI, and IDI, we compared the applicable values between the nomogram and AJCC system. The nomogram-related C-index in the training cohort was greater than the AJCC threshold (Fig. 6). The NRI was 0.21 (95% CI: 0.10–0.49), 0.29 (95% CI: 0.15–0.51), and 0.37 (95% CI: 0.19–0.55) for the 1-, 3-, and 5-year, respectively. The IDI values were 0.10 (95% CI: 0.07–0.15, P < 0.001), 0.12 (95% CI: 0.09–0.17, P < 0.001), and 0.13 (95% CI: 0.08–0.18, P < 0.001) at 1-, 3-, and 5-year, respectively (Table 3).

**Table 3:** NRI and IDI of the nomogram and AJCC staging criteria alone in CSS prediction for postoperative GNEC patients

Index	Training cohort		P value	Validation cohort		P value
	Estimate	95% CI		Estimate	95% CI	
NRI						
For 1-year CSS	0.21	0.10–0.49		0.17	0.03–0.50	
For 3-year CSS	0.29	0.15–0.51		0.311	0.12–0.63	
For 5-year CSS	0.37	0.19–0.55		0.32	0.28–0.67	
IDI						
For 1-year CSS	0.1	0.07–0.15	< 0.001	0.21	0.15–0.31	< 0.001
For 3-year CSS	0.12	0.09–0.17	< 0.001	0.18	0.12–0.26	< 0.001
For 5-year CSS	0.13	0.08–0.18	< 0.001	0.17	0.10–0.25	< 0.001

The outcomes provided compelling evidence that the nomogram outperformed the AJCC stage system regarding application value and predictive ability.

### New Risk Stratification

**Fig. 7:**
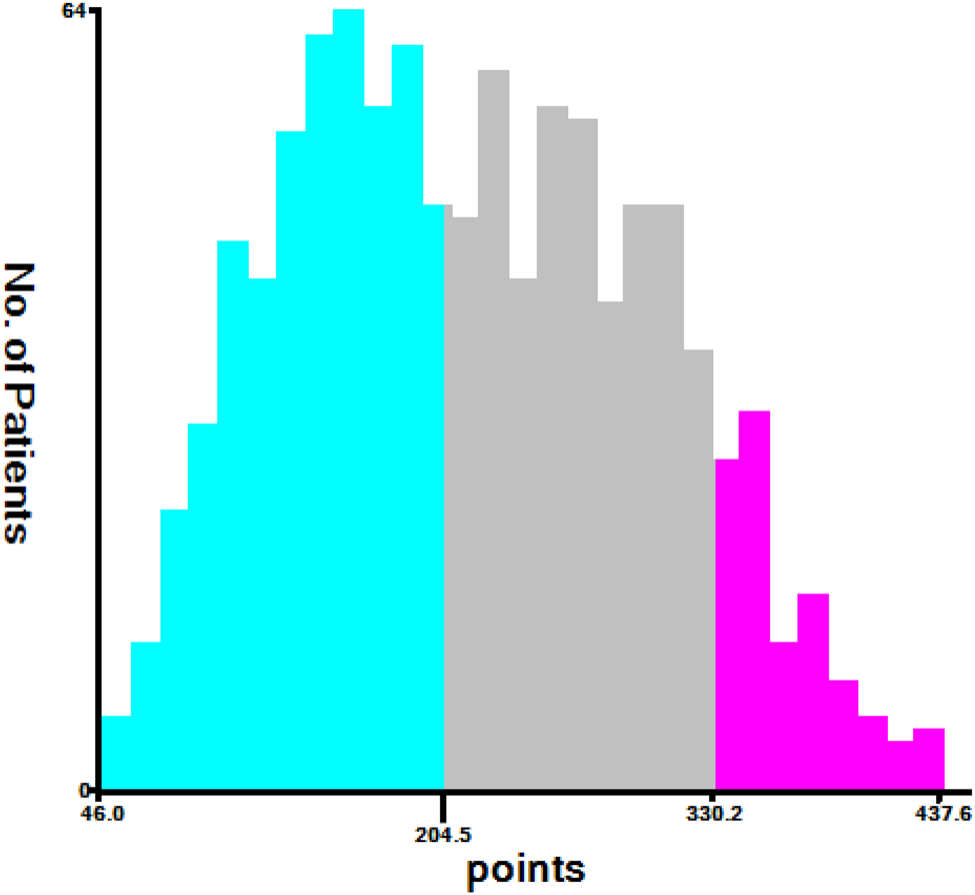
Cut-off point for risk stratification selected using X-tile

**Fig. 8:**
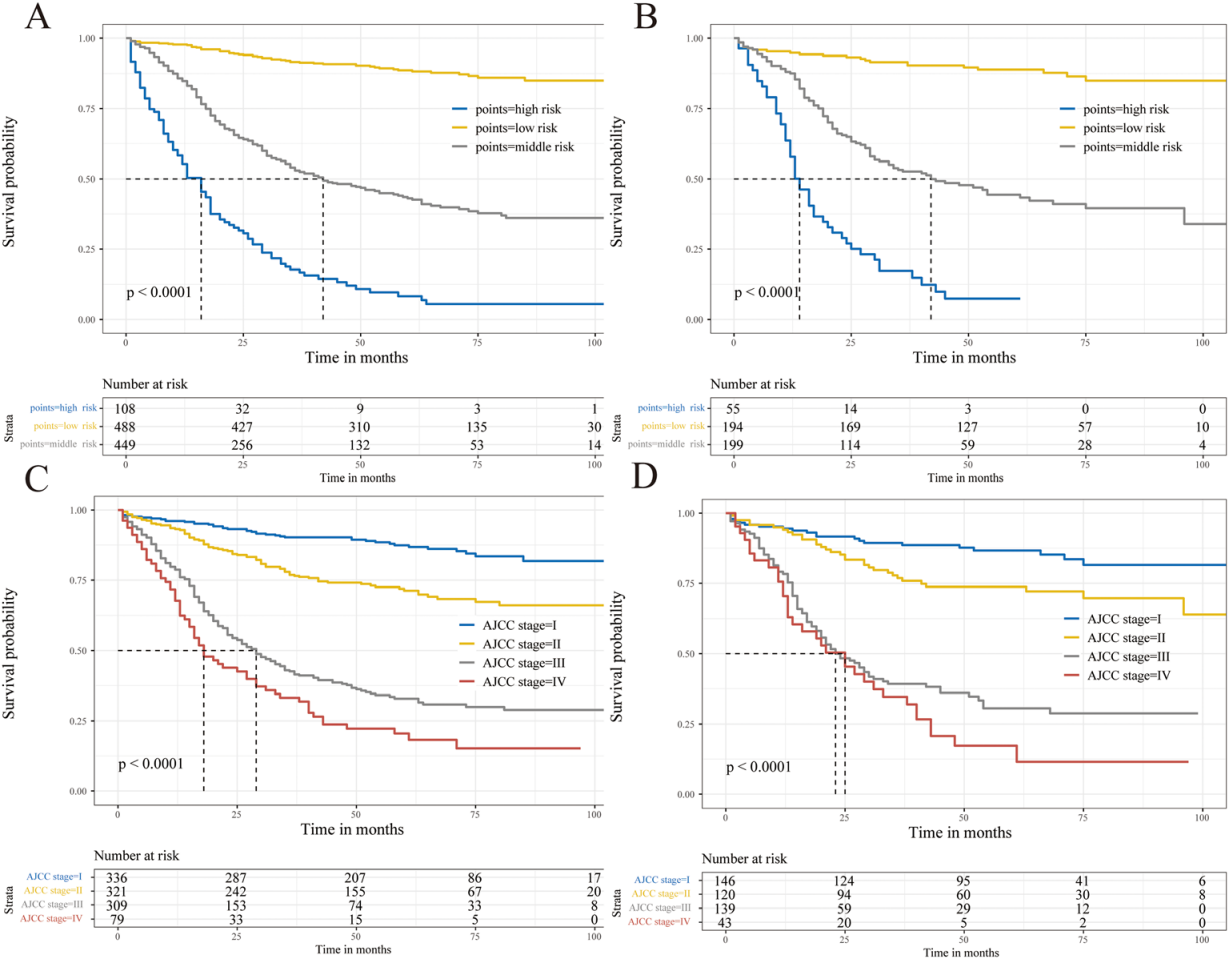
KM analyses of postoperative GNEC patients. **(A, B)** KM curves of training and internal validation cohorts based on the new risk stratification system; **(C, D)** KM curves of training and internal validation cohorts based on AJCC staging criteria

GNEC patients were divided into three risk cohorts based on the examination of the X-tile software: low risk (46.0 < total points < 204.5), middle risk (204.5 < total points < 330.2), and high risk (330.2 < total points < 437.6; Fig. 7). KM curves demonstrated a significant degree of discrimination among the three risk categories (Fig. 8A and B).

Compared with the new risk stratification, the stage I and stage II patients couldn’t be well distinguished in the AJCC staging system. The limited ability to discriminate between stage III and stage IV was also presented (Fig. 8 C and 8D).

### External validation of the nomogram

**Fig. 9:**
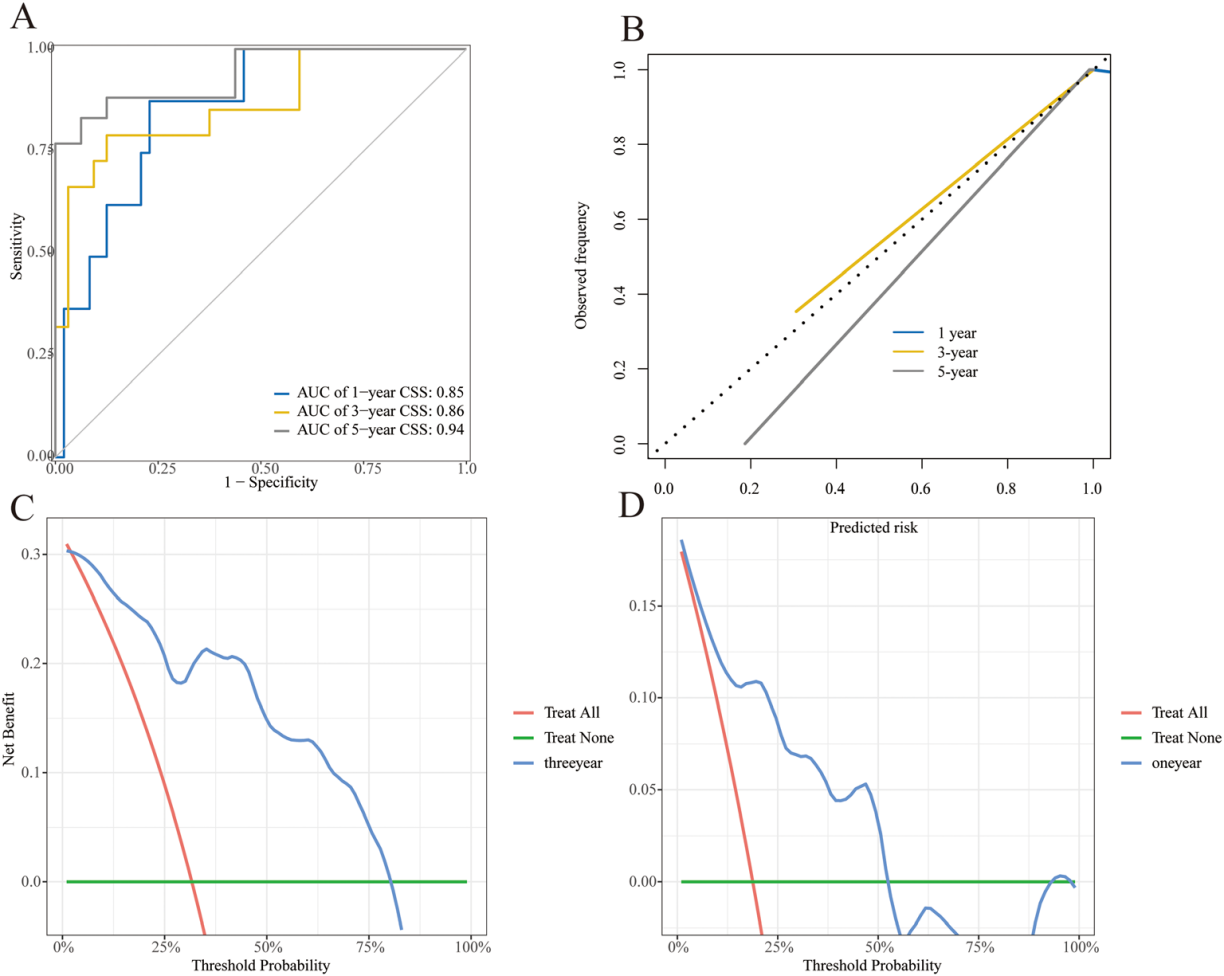
Results of the external validation cohort. **(A)** ROC for 1-, 3-, and 5-year CSS prediction. **(B)** Calibration curves of 1-, 3- and 5-year CSS. **(C, D)** Decision curve analysis of 1-, 3-year CSS

Based on the patient’s data acquired from our institution, external validation was performed to further verify the model’s effectiveness. The ROC curves demonstrated good prediction performance (1-, 3-, and 5-year AUC were 0.85, 0.86, and 0.94, Fig. 9A) of the nomogram. The calibration curves also presented approximate consistency between the predicted and actual results (Fig. 9B). The nomogram’s good clinical practical utility was next confirmed by DCA curves (Fig. 9 C and 9D).

## Discussion

Currently, the low incidence rate and high heterogeneity hindered further investigation of GNEC [15]. Thus, we created and validated a nomogram to predict the 1-, 3-, and 5-year CSS of surgical GNEC patients based on the SEER database. The model demonstrated improved prediction ability compared with the 8th AJCC criteria. We then developed a novel risk stratification system that divided all cases into low-, middle-, and high-risk groups and showed a more remarkable ability to distinguish different risk groups than the conventional AJCC staging system by using X-tile software to determine the cutoff value for the best grouping.

Based on the univariate and multivariate Cox regression analysis, ten parameters (age, gender, grade, T stage, N stage, metastasis, primary site, tumor size, RNE, and chemotherapy) significantly affecting CSS in postoperative GNEC patients were enrolled in the predictive nomogram. By examining the max points of the integrated parameters in the model, grade and TNM stage were regarded as highly significant variables influencing the prognoses. Previous research had shown how these risk variables and GNEC were related. Hu et al. investigated current GNEC epidemiological trends and developed a nomogram to assess these patients’ prognoses. According to the findings, the survival of GNEC was substantially correlated with grade, T, and N staging [12]. Another Chinese study examined the features of 132 Chinese GNEC patients and found that the patients’ survival was independently predicted by the size of the tumor, N stage, mitotic index, radical surgery, and adjuvant treatment [7]. Xu et al. conducted a study comparing intestinal-type GC (IGC) and GNEC patients, finding that GNEC patients had a better prognosis than ICG in the early stages of the tumor. Furthermore, age, gender, tumor size, AJCC stage, T stage, N stage, and surgery were all substantially associated with the overall survival of GNEC patients [16]. Concordantly, the Cox regression analysis proposed in our study also identified old age, gender of male, larger tumor size, and higher TNM stage as independent risk variables for CSS. Larger tumor size and higher TNM stage usually indicated more aggressive tumor behavior with worse survival. Age was also a risk factor, which might be attributed to the factor that elderly patients had worse general conditions and suffered more chronic diseases. The nomogram model included risk factors that were easily gotten and collected from the patient’s hospitalization information. By using the variable score, clinicians could predict the prognosis accurately and evaluate the treatment benefits. This provided a visual means of communication between clinicians and patients. Through the nomogram chart, patients could know their own probability of survival, which would prompt them to make more reasonable treatment choices. Besides, several variables with higher scores, such as older age and gender of male, should be concerned for the relatively poor prognosis.

It was reported that GNEC patients presented irresponsiveness to traditional chemotherapy [17]. But based on our analysis, chemotherapy could improve postoperative survival among GNEC after surgical resection, indicating that adjuvant chemotherapy should still be adopted. In China, patients with GNEC were typically treated with the same chemotherapy agents as those with gastric adenocarcinoma, including fluorouracil, cisplatin, streptomycin, allium annulus, and paclitaxel [18, 19]. Therefore, specific chemotherapeutic drugs and regimens for GNEC should be further explored. In addition, as shown in the model, the primary site of cardia was correlated with poorer prognoses. GC could be divided into cardia and non-cardia according to the tumor location, which presented different epidemiology and tumor behavior [20]. And GC patients with cardia invasion tended to suffer worse survival in previous studies [21]. So, our research first validated that the primary site of cardia was a risk factor in postoperative GNEC patients.

Currently, the AJCC TNM staging system is the main option for cancer prognosis prediction. However, its application in GNEC patients has recently been called into question [7]. It was reported that other clinical parameters, such as gender, grade, and adjuvant therapy, were significantly related to the prognoses of GNEC patients [22–24]. So, we created a nomogram by combining various factors (such as demographic and clinicopathologic traits) affecting CSS in patients with GNEC. Additionally, depending on their overall scores, patients were categorized into low-, middle-, and high-risk groups. On this premise, it was possible to compare the power of the nomogram with the conventional AJCC staging system, which was not performed in the previous research. The nomogram demonstrated better predictive ability than tumor staging based solely on AJCC criteria, according to the NRI, IDI, and C-index data. Besides, the effectiveness of the predictive model was tested in both the internal validation and external validation cohorts. According to the KM curves, the stage I and stage II patients couldn’t be well distinguished in the AJCC staging system. The limited ability to discriminate between stage III and stage IV was also presented. Surprisingly, compared to the conventional staging system, the KM analysis showed significantly different CSS among the three risk groups, which can help clinicians customize their management and treatment plans.

### Limitation

The current study included various limitations due to the nature of the database, which should be considered when interpreting the results. To begin, because this was a retrospective study, selection bias was unavoidable. Second, the SEER database lacked information on precise data on performance status, comorbidities, varied chemotherapy regimens, treatment for distant metastases, somatostatin analogues, and “neuroendocrine cancer-specific” data. Furthermore, the SEER database’s GNEC classification was not completely the same as the WHO classification. Finally, even though the research was externally validated, the small sample data could only provide limited support for the result. Multicenter data with a larger sample size are required to further evaluate the predictive model.

## Conclusion

In summary, we constructed and validated a nomogram to predict 1-, 3-, and 5-year CSS for GNEC patients after surgical resection. The prognostic factors identified in the model could help doctors better understand the rare malignancy and enhance the treatment plan. Larger and multicenter samples are required to assess and validate its performance and broaden its applicability.

## Data Availability

The datasets created and evaluated during this project are housed in the SEER database (https://seer.cancer.gov/). Yafang Lou could be reached for study data upon reasonable request.
